# A Nomenclature for Vertebral Fossae in Sauropods and Other Saurischian Dinosaurs

**DOI:** 10.1371/journal.pone.0017114

**Published:** 2011-02-28

**Authors:** Jeffrey A. Wilson, Michael D. D'Emic, Takehito Ikejiri, Emile M. Moacdieh, John A. Whitlock

**Affiliations:** Museum of Paleontology and Department of Geological Sciences, University of Michigan, Ann Arbor, Michigan, United States of America; Raymond M. Alf Museum of Paleontology, United States of America

## Abstract

**Background:**

The axial skeleton of extinct saurischian dinosaurs (i.e., theropods, sauropodomorphs), like living birds, was pneumatized by epithelial outpocketings of the respiratory system. Pneumatic signatures in the vertebral column of fossil saurischians include complex branching chambers within the bone (internal pneumaticity) and large chambers visible externally that are bounded by neural arch laminae (external pneumaticity). Although general aspects of internal pneumaticity are synapomorphic for saurischian subgroups, the individual internal pneumatic spaces cannot be homologized across species or even along the vertebral column, due to their variability and absence of topographical landmarks. External pneumatic structures, in contrast, are defined by ready topological landmarks (vertebral laminae), but no consistent nomenclatural system exists. This deficiency has fostered confusion and limited their use as character data in phylogenetic analysis.

**Methodology/Principal Findings:**

We present a simple system for naming external neural arch fossae that parallels the one developed for the vertebral laminae that bound them. The nomenclatural system identifies fossae by pointing to reference landmarks (e.g., neural spine, centrum, costal articulations, zygapophyses). We standardize the naming process by creating tripartite names from “primary landmarks,” which form the zygodiapophyseal table, “secondary landmarks,” which orient with respect to that table, and “tertiary landmarks,” which further delineate a given fossa.

**Conclusions/Significance:**

The proposed nomenclatural system for lamina-bounded fossae adds clarity to descriptions of complex vertebrae and allows these structures to be sourced as character data for phylogenetic analyses. These anatomical terms denote potentially homologous pneumatic structures within Saurischia, but they could be applied to any vertebrate with vertebral laminae that enclose spaces, regardless of their developmental origin or phylogenetic distribution.

## Introduction

Living archosaurs (i.e., birds and crocodylians) are characterized by the presence of pneumatic outpocketings of the respiratory epithelium that invade certain bones. Cranial skeletal pneumaticity is present in both crocodylians and birds [1], as well as their common ancestor and many of its descendants [2]. Postcranial skeletal pneumaticity, in contrast, is restricted to birds among living archosaurs [3]. Among fossil archosaurs, postcranial skeletal pneumaticity is present in bird-line archosaurs (i.e., Ornithodira), and it may have been present in some [3] or many [4] crocodile-line archosaurs. Postcranial pneumaticity is most typically manifest in the axial skeleton of ornithodirans, although appendicular bones are also pneumatized in volant forms (i.e., pterosaurs, birds) and their close relatives [5], [6].

**Figure 1:**
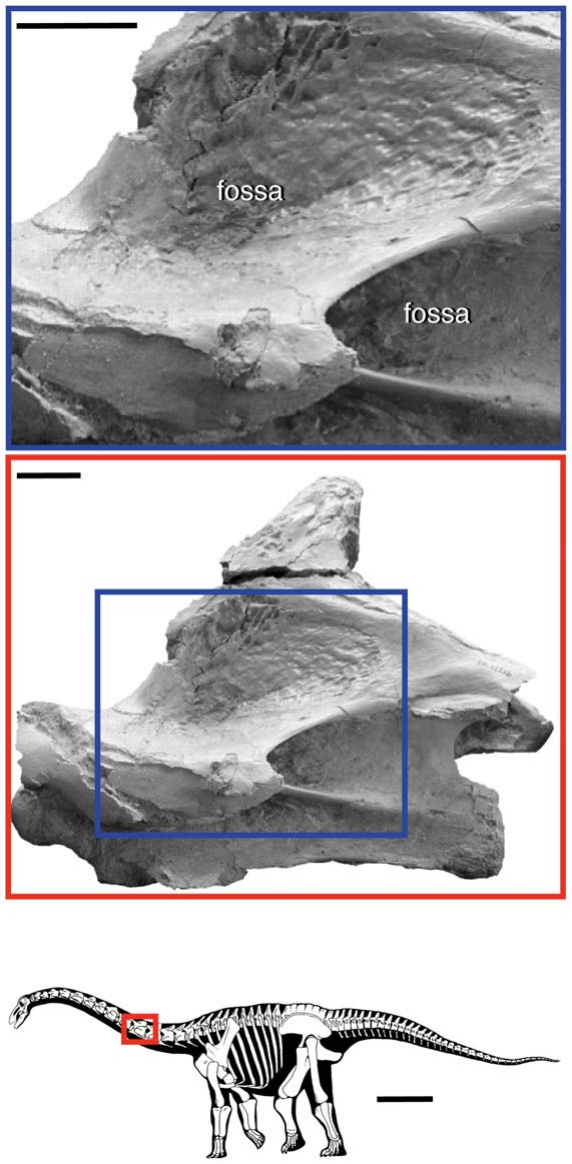
Vertebral fossae in the sauropod dinosaur *Rapetosaurus krausei*. Vertebral fossae in sauropods are hypothesized to be produced by pneumaticity, which is usually limited to the axial column, excluding the atlas, chevrons, and distal caudal vertebrae (bottom image). The middle photograph shows fossae in a cervical vertebra, which in the neural arch are bounded by vertebral laminae. The close-up photograph (top) shows the that bone texture within the fossa is often smooth, crenulated, and shiny, which is indicative of pneumatic bone. Silhouette reconstruction from [49]; cervical vertebra from [59]:fig. 10). Scale bar equals 1 m in silhouette; scale bars equal 3 cm in photographs. ©Copyright 2009 The Society of Vertebrate Paleontology. Reprinted and distributed with permission of the Society of Vertebrate Paleontology.

Among non-volant ornithodirans, axial pneumaticity is perhaps best developed in sauropod dinosaurs, in which pneumatic diverticulae leave their traces in postatlantal vertebrae and ribs, but apparently not chevrons (Fig. 1). Axial pneumaticity can take the form of deep and sometimes complex invasion of internal bone, or in the form of spaces enclosed by bony laminae connecting the processes projecting from the neural arch. The former, which we refer to as “internal pneumaticity,” displays variation that appears to characterize sauropod subgroups [7] and has important implications for sauropod paleobiology [8]. Internal pneumatic structures are typically not bounded by landmarks, however, and it is very difficult to homologize individual pneumatic spaces between vertebrae or between species. Neural arch fossae, on the other hand, are typically bounded by vertebral laminae and easily homologized within and between taxa. These structures display important phylogenetic variation that has not been extensively sampled thus far. We provide a practical nomenclature for lamina-bounded fossae that takes advantage of conventions and landmarks used in existing nomenclature for vertebral laminae [9]. The nomenclature for lamina-bounded fossae is designed to facilitate their use in comparative anatomy and phylogenetic analysis.

### Neural arch fossae

Our nomenclature applies to cavities or fossae bounded by vertebral laminae, which in sauropods and other saurischians are likely formed by, or in concert with, pneumatic diverticulae [10], [11]. Fossae may also contain ligamentous attachment sites in addition to any pneumatic structures. In other vertebrates, vertebral laminae and fossae may not be associated with pneumaticity, but the proposed terminology still applies. Multiple terms have been used to describe these features, such as “cavity” [12]; “chonos” [13], and “coel” [5], but in keeping with recent work on pneumaticity in bird-line archosaurs [3], we adopt the more general term “fossa.” Pneumatic fossae may be sharp-lipped, and the surface of the concavity may bear a distinctive smooth, shiny, or crenulated texture ([11]; Fig. 1). In contrast, pneumatic fossae in the centrum are not bounded by landmarks and are excluded from the nomenclatural system presented here. They may be referred to as pneumatic fossae, pneumatic foramina, or ‘pleurocoels.’

Our nomenclature is designed for sauropod dinosaurs, which exhibit a highly complicated system of neural arch fossae. This nomenclature is applicable to other ornithodirans with vertebral pneumatic fossae (e.g., theropods, pterosaurs), as well as to tetrapods with morphologically and topologically similar cavities bounded by laminae in their vertebrae, regardless of whether they are thought to be pneumatic (e.g., the hadrosaurid *Gryposaurus* [14], the rauisuchian *Postosuchus* [15], the phytosaur *Machaeroprosopus* [16]).

### Rationale

Numerous descriptive studies of saurischian vertebrae have employed terms for specific fossae on the centrum and neural arch of saurischian vertebrae. These terminologies vary in their comprehensiveness, with some naming most or all fossae on the vertebrae of a given species [12], [13], [17]–[19] and others referring to particular fossae of interest [20]–[23]. As discussed below, these terms cannot easily be adapted into a comprehensive, landmark-based nomenclatural system that is simple, intuitive, and scalable. Nonetheless, these studies include many novel observations and establish useful conventions, some of which we adopt in our proposed system.

In his description of the theropod *Dilophosaurus wetherilli*, Welles [13] created a series of terms to describe each conical depression, or “chonos” (*Gr*. “funnel”), associated with the diapophysis and zygapophyses (Table 1). He also established a vertebral plane, which he called the “table,” that provided an orientational reference. The resultant terms he created are partially landmark based, typically relying on a single vertebral process (e.g., the prezygapophysis) and an orientational descriptor (e.g., “pre”, “post”; above or below table). The terms exhaustively describe the neural arch fossae of *Dilophosaurus* and were later applied to *Ceratosaurus* [24], but they are not easily applied to vertebrae that have more complicated patterns of laminae and fossae, because several fossae may be present in the area described by a landmark and orientational descriptor.

**Table 1:** Comparison of nomenclature for neural arch fossae.

	Hatcher 1901(8 cavities)	Osborn & Mook 1921(7 cavities)	Welles 1984(9 chonoses)	Bonaparte 1999(11 cavities)	Harris 2006(12 fossae)	this paper(16 fossae)
**D**	infradiapophyseal cavity	infradiapophyseal cavity	medial chonos	central infradiapophyseal cavity	infradiapophyseal fossa	centrodiapophyseal fossa (cdf)
	prediapophyseal cavity	infraprezygapophyseal cavity	anterior chonos	anterior infradiapophyseal cavity	cranial infradiapophyseal fossa	prezygapophyseal centrodiapophyseal fossa (prcdf)
	postdiapophyseal cavity	infrapostzygapophyseal cavity	posterior chonos	posterior infradiapophyseal cavity; infrapostzygapophyseal depression	caudal infradiapophyseal fossa	postzygapophyseal centrodiapophyseal fossa (pocdf)
	—	—	—	—	infradiapophyseal fossa	parapophyseal centrodiapophyseal fossa (pacdf)
	—	—	—	postparapophyseal fossa	cranial infradiapophyseal fossa	prezygapophyseal parapodiapophyseal fossa (prpadf)
	supradiapophyseal cavity	—	—	depression lateral to the diapophyseal lamina	parazygapophyseal fossa	spinodiapophyseal fossa (sdf)
	—	supraprezygapophyseal cavity	—	—	—	prezygapophyseal spinodiapophyseal fossa (prsdf)
	—	suprapostzygapophyseal cavity	—	—	paraspinous fossa	postzygapophyseal spinodiapophyseal fossa (posdf)
	—	supradiapophyseal cavity	—	depression of the diapophyseal lamina	—	spinodiapophyseal fossa 1 (sdf1)
	—	—	—	—	—	spinodiapophyseal fossa 2 (sdf2)
**PA**	—	—	—	postparapophyseal fossa	infraparapophyseal fossa	centroparapophyseal fossa (cpaf)
**PR**	infraprezygapophyseal cavity	—	prechonos	circumneural cavity; supraneural cavity	cranial infrazygapophyseal fossa	centroprezygapophyseal fossa (cprf)
	—	—	—	—	—	parapophyseal centroprezygapophyseal fossa (pacprf)
	supraprezygapophyseal cavity	—	prespinal chonos	prespinal cavity	prespinous fossa + cranial elastic ligament fossa	spinoprezygapophyseal fossa (sprf)
**PO**	infrapostzygapophyseal cavity	—	postchonos	circumneural cavity; supraneural cavity	caudal infrazygapophyseal fossa	centropostzygapophyseal fossa (cpof)
	suprapostzygapophyseal cavity	postspinal cavity	postspinal chonos	postspinal cavity	postspinous fossa + caudal elastic ligament fossa	spinopostzygapophyseal fossa (spof)

For convenience, the table has been organized anatomically into diapophyseal (D), parapophyseal (PA), prezygapophyseal (PR), and postzygapophyseal (PO) fossae. Within each of these categories, central fossae are listed before spinal fossae. In some cases, previous authors did not specify a name for a fossa that we name here (marked with a “—”); in other cases, authors use the same term for fossae that we give different names to. Hatcher (1901∶18) also mentioned “spinal cavities”, which are small, irregular pockets in the laminae of the neural spine. These are not landmark-bounded fossae and are not named here. Welles (1984) also mentioned a “lateral chonos”, but it is not clear to us how that fossa differs from the medial chonos, so we didn't include it in that table. The nomenclature of Bonaparte (1999) has been translated from the Spanish. The “angular cavity” of Bonaparte (1999) was not included here because it appears to name a fossa within divided lamina (cpol). Note that the distinction between the supraneural and circumneural cavities of Bonaparte (1999) is not clear.

Early terminology [12], [17], as well as later iterations [18], [19], also typically employ a single landmark and an orientational descriptor (Table 1). There have been numerous terms applied to fossae in the neural arch of sauropod vertebrae, which has resulted in confusion. Despite the proliferation of terms, no system has emerged from them, and none of the terms listed in Table 1 has gained primacy or currency in the literature.

Previous sets of terms are problematic for several reasons. Some terms are ambiguous because more than one fossa is present in the area pointed to by the landmark and orientation. Although in some cases this can be an appropriate way to describe morphology (i.e., bipartite vs. tripartite naming; see “Practical Application” below), in most cases it is imprecise. Examples of ambiguous terms include the “infradiapophyseal fossa” and “peduncular fossa” (e.g., [12], [25]). Other terms are misleading because different authors use the same term to refer to different structures. Additionally, the proliferation of names has resulted in the same structure being named and renamed repeatedly, in some cases as many as five times (Table 1). In other cases, differences of opinion lead to terminological confusion. Certain authors may apply a single name to a structure that other authors interpret as including two structures, each deserving of their own name, and vice versa.

Both inadvertent and intentional disagreement about the terms applied to neural arch fossae can lead to missed opportunities to recognize potentially homologous structures [26]. This leads us to propose a new system that reuses many conventions but nonetheless introduces new terms. By creating a flexible, comprehensive, and intuitive system, we hope not only to simplify the work of comparative anatomists, but also to systematize the naming process.

## Materials and Methods

The nomenclatural system for vertebral fossae we propose here is based on our combined collections research at the institutions listed below. For the anatomical structures listed below and discussed in text, we use “Romerian” terms [27] for the structures (e.g., “centrum,” not “corpus”) and for their orientation (e.g., “anterior,” not “cranial”).

### Institutional Abbreviations

**AMNH**, American Museum of Natural History, New York, USA; **CM**, Carnegie Museum of Natural History, Pittsburgh, USA; **CSPGM**, Collections Paléontologiques du Service Géologique du Maroc, Rabat, Morocco; **FMNH**, Field Museum of Natural History, Chicago, USA; **IGM**, Geological Institute of the Mongolian Academy of Sciences, Ulaanbaatar, Mongolia; **MACN**, Museo Argentino de Ciencias Naturales “Bernardo Rivadavia”, Buenos Aires, Argentina; **MB.R.**, Humboldt Museum für Naturkunde, Berlin, Germany; **MNHN**, Muséum National d'Histoire Naturelle, Paris, France; **MNN**, Museé National du Niger, Niamey, Niger; **MUCP**, Museo de la Universidad Nacional del Comahue, Neuquén, Argentina; **NSMT**, National Science Museum, Tokyo, Japan; **ZDM**, Zigong Dinosaur Museum, Zigong, China.

### Anatomical Abbreviations

**acdl**, anterior centrodiapophyseal lamina; **acpl**, anterior centroparapophyseal lamina; **c**, centrum; **ca**, caudal vertebra; **cdf**, centrodiapophyseal fossa; **cpol**, centropostzygapophyseal lamina; **cpol-f**, centropostzygapophyseal lamina fossa; **cprl**, centroprezygapophyseal lamina; **cprl-f,** centroprezygapophyseal lamina fossa; **cv**, cervical vertebra; **d**, diapophysis; **dv**, dorsal vertebra; **eprl**, epipophyseal-prezygapophyseal lamina; **pa**, parapophysis; **pacdf**, parapophyseal centrodiapophyseal fossa; **pacprf**, parapophyseal centroprezygapophyseal fossa; **pcpl**, posterior centroparapophyseal lamina; **po**, postzygapophysis; **pocdf**, postzygapophyseal centrodiapophyseal fossa; **podl**, postzygodiapophyseal lamina; **posdf**, postzygapophyseal spinodiapophyseal fossa; **posl**, postspinal lamina; **ppdl**, paradiapophyseal lamina; **pr**, prezygapophysis; **prcdf**, prezygapophyseal centrodiapophyseal fossa; **prdl**, prezygodiapophyseal lamina; **prdl-f**, prezygodiapophyseal lamina fossa; **prpadf**, prezygapophyseal paradiapophyseal fossa; **prsdf**, prezygapophyseal spinodiapophyseal fossa; **prsl**, prespinal lamina; **s**, neural spine; **sdf**, spinodiapophyseal fossa; **spdl**, spinodiapophyseal lamina; **spol**, spinopostzygapophyseal lamina; **spol-f**, spinopostzygapophyseal lamina fossa; **sprl**, spinoprezygapophyseal lamina.

## Results

### Nomenclatural System for Neural Arch Fossae

Most neural arch fossae can be defined by the vertebral laminae that enclose them, and the most informative nomenclatural system would employ those laminae in the name for that fossa. For example, a fossa delimited by the postzygodiapophyseal lamina (podl), the spinopostzygapophyseal lamina (spol), and the spinodiapophyseal lamina (spdl) could receive a name that is a combination of these three names or their abbreviations. Such a nomenclatural system, although maximally informative, would not be practical because the names would be cumbersome and inefficient (i.e., “postzygodiapophyseal-spinopostzygapophyseal-spinodiapophyseal fossa”). If abbreviations for laminae are used instead, the resultant name for the fossa is shorter but no more pronounceable (“podl-spol-spdl fossa”), even if redundant letters are removed (“pod-spo-spd fossa”). The problem with naming fossae by their laminae, whether in full or abbreviated, is that it creates names that contain duplicate information. In the examples above, the combinative forms “spino”, “diapo”, and “postzygo”, or their respective abbreviations, each appear twice. This redundancy is inherent, because vertebral laminae are landmarks whose names are themselves based on landmarks.

We propose a simple nomenclatural system that constructs names for fossae based on the same landmarks that define laminae. As such, the proposed system for naming fossae parallels that developed for naming vertebral laminae [9]. In the example above, the fossa enclosed by the postzygodiapophyseal, spinopostzygapophyseal, and spinodiapophyseal laminae would be named on the basis of the postzygapophysis, diapophysis and spine — that is, by the vertices of the fossa rather than by its sides (i.e., the laminae). Because there is no inherent order for combining these three terms or any three terms that define a fossa, we establish an arbitrary set of three “primary landmarks” that form a reference plane, “secondary landmarks” that specify the fossa's position with respect to the reference plane, and “tertiary landmarks” that further distinguish the fossa from other neighboring fossae.

### Landmarks and the Zygodiapophyseal Table

**Figure 2:**
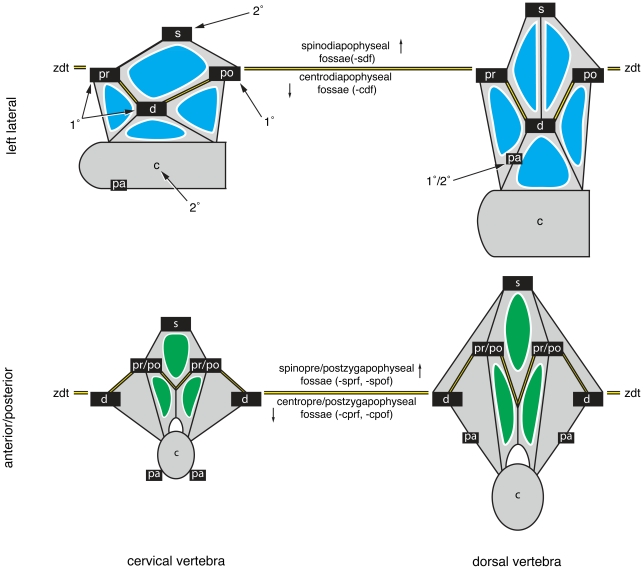
Primary landmarks, secondary landmarks, and the zygodiapophyseal table. Schematic diagrams of a cervical vertebra (left) and dorsal vertebra (right) in left lateral view (top) and anterior/posterior view (bottom). The zygodiapophyseal table (zgt) is formed by the primary landmarks (1°): the prezygapophysis (pr), postzygapophysis (po), and diapophysis (d). The zygodiapophyseal table is indicated by the double black lines highlighted in yellow. The neural spine (s) and centrum (c) are secondary landmarks (2°) that orient with respect to zygodiapophyseal table. In middle and posterior dorsal vertebrae, the parapophysis (pa) can act as either a primary or secondary landmark (see “Practical Application” for details). Diapophyseal fossae are in blue, and pre/postzygapophyseal fossae are in green.

Historically, students of dinosaur vertebral anatomy have referred to fossae appearing above and below the zygapophyses and diapophysis (e.g., “infradiapophyseal fossa” [17]; Table 1). That is, these students used the plane, or “table”, formed by these processes to orient fossae [13]. For reasons discussed above, orientational descriptors and a single landmark are not always sufficient to point to a specific fossa, but we nonetheless adapt this historical practice to the proposed system. This we do by reference to one of three “primary landmarks” that define the zygodiapophyseal table and reference to a “secondary landmark” that orients with respect to it. The diapophyses (d), prezygapophyses (pr), and postzygapophyses (po) define the zygodiapophyseal table and are here arbitrarily referred to as “primary landmarks” because in our system they take primacy in the name for the fossa (e.g., a “diapophyseal fossa” or “df”). Because a given fossa may be bounded by two of the three primary landmarks, we arbitrarily define the diapophysis as the ‘primary’ primary landmark. As a rule of thumb, fossae visible in lateral view are typically diapophyseal fossae, whereas those visible in anterior or posterior views are prezygapophyseal or postzygapophyseal fossae, respectively (Fig. 2). The neural spine (s), centrum (c), and occasionally the parapophysis (pa) act as “secondary landmarks” that indicate the position of the fossa above or below the zygodiapophyseal plane (see “Practical Application”). Together, primary and secondary landmarks form a bipartite name. A diapophyseal fossa that is also bounded by the centrum is a “centrodiapophyseal fossa” or “cdf”; a postzygapophyseal fossa that is also bounded by the neural spine is a “spinopostzygapophyseal fossa” or “spof”. Bipartite names typically refer to a set of fossae, although there are cases when they can refer to a single fossa (see below). A “tertiary landmark” provides the final point of reference for a named fossa by discriminating within a set of fossae. The tertiary landmark is added to the front of any bipartite name to form a tripartite name (e.g., “prezygapophyseal centrodiapophyseal fossa” or “prcdf”). There are only three possible tertiary landmarks, the parapophysis (pa), prezygapophysis (pr), and postzygapophysis (po). The diapophysis, centrum and neural spine cannot act as tertiary landmarks because a landmark can only be used once to define a fossa (i.e., no “diapophyseal spinodiapophyseal fossa”). Any fossa bounded by the diapophysis would be a diapophyseal fossa, and any fossa bounded by the neural spine or centrum would have them already employed as secondary landmarks.

Both bipartite and tripartite names can easily be distilled into five- or six-letter abbreviations for use in figures or discussion in text. Following conventions developed for vertebral laminae, landmarks can be represented as single- or double-letter abbreviations: c, centrum; d, diapophysis; pa, parapophysis; po, postzygapophysis; pr, prezygapophysis; s, neural spine. Abbreviated names for fossae are constructed from a tertiary landmark (if required) placed in front of a bipartite name constructed from a secondary and primary landmark.

### Practical Application

The nomenclatural system we propose names only those fossae that are bounded by the primary, secondary, and usually tertiary landmarks, as well as fossae within laminae and those associated with the eprl (see “Special Cases”). Names are not applied to fossae that are bounded solely by other landmarks (e.g., unnamed vertebral laminae) or those not bounded by landmarks at all (e.g., irregular fossae; fossae in the centrum).

**Figure 3:**
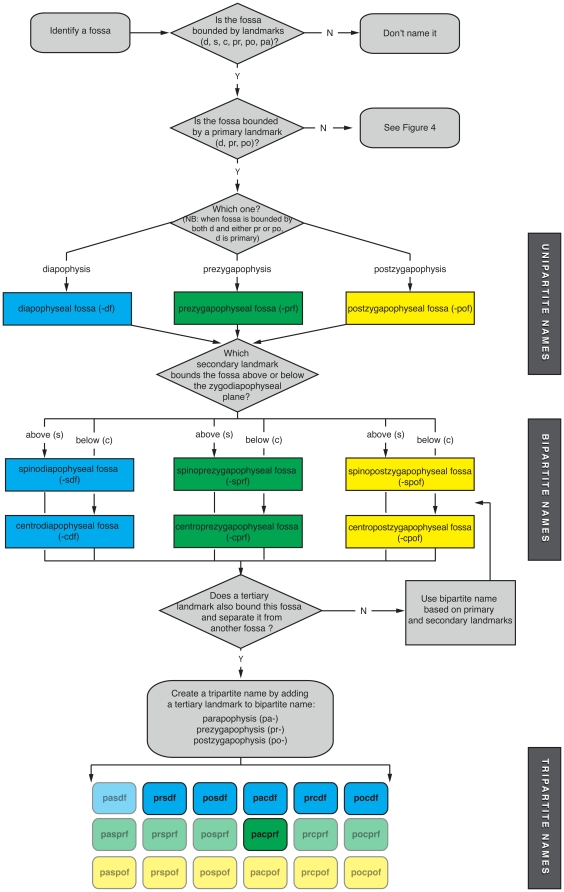
Flowchart explaining the construction of simple bipartite and tripartite names for fossae on neural arches. These decision trees show how to name fossae. Rounded rectangles are starting/stopping points, and diamonds represent decisions. Starting from the upper left, primary, secondary, and tertiary landmarks are identified in succession. The majority of landmark-bounded fossae can be identified by one of the tripartite names created by combining one of three primary landmarks (diapophysis, prezygapophysis, postzygapophysis), one of two secondary landmarks (neural spine, centrum), and one of three tertiary landmarks (parapophysis, prezygapophysis, postzygapophysis). The resultant named fossae recognized here are shown at the bottom of the flowchart. Diapophyseal fossae are in blue, prezygapophyseal fossae are in green, and postzygapophyseal fossae are in yellow. The fossae that are not possible because they involve landmarks at opposite ends of the vertebra (e.g., prcpof, paspof) are rendered semi-transparent.

The process for naming most neural arch fossae is illustrated in the flowchart in Figure 3. A named fossa must be defined by two or three landmarks and receive a bipartite or tripartite name, respectively. Primary, secondary, and tertiary landmarks are identified sequentially. The primary landmark can be thought of as indicating which neural arch surfaces the fossa occupies: lateral (-df), anterior (-prf), or posterior (-pof). Secondary landmarks further localize the fossa in one of six subregions on the neural arch (-sdf, -cdf; -sprf, -cprf; -spof, -cpof). In some cases, a single fossa occupies the entire subregion and receives a bipartite name. Most fossae, however, require a tertiary landmark to be distinguished from others. Theoretically, any of the six bipartite names can be modified by any of three tertiary landmarks (pa-, pr-, po-), but several names are not observed in fossil saurischians due to the relative positions of the landmarks (e.g., “prezygapophyseal spinopostzygapophyseal fossa,” “parapophyseal spinodiapophyseal fossa”). This leaves six bipartite names and six tripartite names for fossae based on the three primary landmarks, two secondary landmarks and three tertiary landmarks.

**Figure 4:**
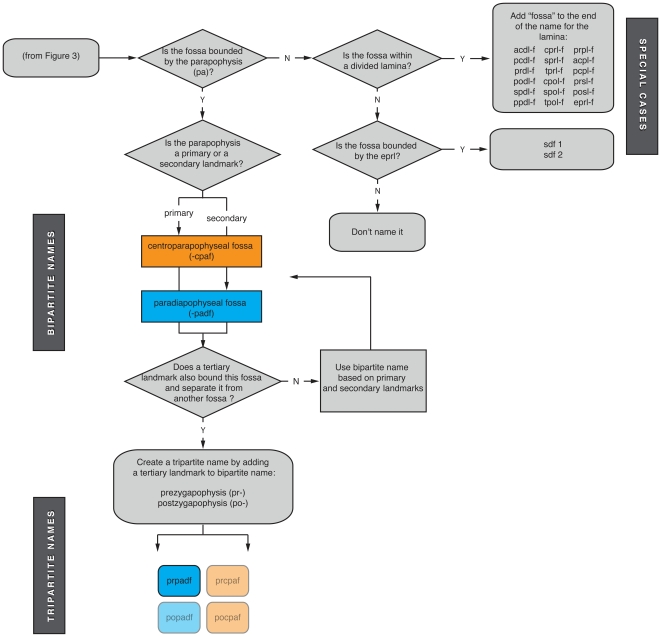
Flowchart explaining the construction of names for fossae associated with the parapophysis, divided laminae, and the eprl. These decision trees show how to name fossae. Rounded rectangles are starting/stopping points, and diamonds represent decisions. Pneumatic fossae associated with the parapophysis In dorsal vertebrae can act as either a primary or secondary landmarks (see text for explanation). Sets of bipartite and tripartite names associated with the parapophysis are shown at left. Diapophyseal fossae are in blue, and parapophyseal fossae are in orange. “Impossible” fossae are rendered semi-transparent. At right are shown fossae associated with divided laminae and the spinodiapophyseal fossa (sdf).

**Figure 5:**
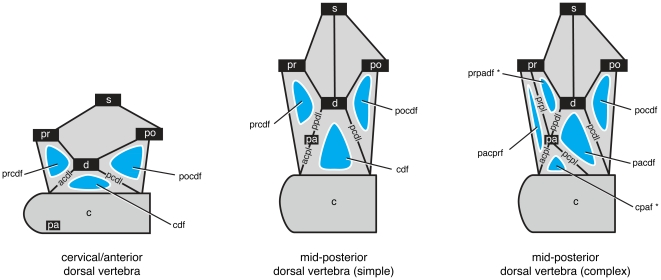
Configuration of vertebral laminae and fossae associated with the parapophysis in presacral vertebrae. Left, a cervical or anterior dorsal vertebra, in which the parapophysis is positioned on the centrum. Two laminae extend ventrally from the diapophysis (acdl, pcdl), helping to bound three fossae (prcdf, cdf, pocdf). Center, a simple mid- or posterior dorsal vertebra in which the parapophysis has risen onto the neural arch and is connected to the diapophysis and anterior centrum via two complementary laminae (ppdl, acpl). The configuration of fossae and their nomenclature, however, remains the same: the three fossae are still bounded by the zygapophyses, diapophysis, and centrum. Right, a complex mid- or posterior dorsal vertebra in which the parapophysis has risen onto the neural arch and four, rather than two, laminae extend from it (ppdl, acpl, pcpl, prpl). The addition of two laminae bisects the fossae between the diapophysis and centrum (cdf) and between the diapophysis, centrum, and prezygapophysis (prcdf). Four fossae are created, two of which require special naming (noted by asterisks). The fossa between the parapophysis and centrum does not contact the zygodiapophyseal table and thus lacks a primary landmark; in this case the parapophysis is enlisted as a primary landmark (cpaf). The fossa between the diapophysis, parapophysis, and prezygapophysis is not bounded by a secondary landmark (i.e., neural spine or centrum), and the parapophysis is enlisted as a secondary landmark (prpadf).

These six bipartite names and six tripartite names serve to identify the vast majority of neural arch fossae, but fossae associated with the parapophysis require further explication (Fig. 4). In cervical and anterior dorsal vertebrae, the parapophysis is situated on the centrum and there are only two laminae emanating ventrally from the diapophysis: the anterior and posterior centrodiapophyseal laminae (acdl, pcdl). Together with centrozygapophyseal laminae (cprl, cpol) and zygodiapophyseal laminae (prdl, podl), the acdl and pcdl bound three fossae, the prcdf, cdf, and pocdf (Fig. 5). In middle and posterior dorsal vertebrae, the parapophysis migrates onto the neural arch in the path of the acdl, essentially breaking it into complementary laminae known as the parapodiapophyseal lamina (ppdl) and the anterior centroparapophyseal lamina (acpl). On its own, this change in laminar configuration does not alter the configuration of the two fossae associated with the the diapophysis, centrum, and prezygapophysis (Fig. 5). However, in most cases the parapophysis develops its own laminae that connect to the prezygapophysis (i.e., the prpl) and to the centrum (i.e., the pcpl) and subdivide the prcdf and cdf, respectively (Fig. 5). Of the four resultant fossae, two do not fit into the system of 12 bipartite and tripartite fossae described above: one does not contact the zygodiapophyseal table (and thus the primary landmarks), and the other contacts the zygodiapophyseal table but does not contact a secondary landmark. To accommodate this special case, we allow the parapophysis to act as a primary landmark (-paf) in the former case and a secondary landmark (-padf) in the latter case (Fig. 4). Two bipartite fossae (cpaf, padf) and one tripartite fossa (prpadf), constructed using the parapophysis as primary and secondary landmarks, are recognized here. This yields a total of eight bipartite and seven tripartite names that describe all vertebral fossae of sauropods with the exception of the special cases that we describe below.

### Special Cases

The nomenclature outlined above covers the vast majority of neural arch fossa present in sauropods and other saurischians, but there are two special cases that warrant additional discussion: division of the spinodiapophyseal fossa by the epipophyseal-prezygapophyseal lamina and fossae within divided laminae.

#### The epipophyseal-prezygapophyseal lamina and the spinodiapophyseal fossa

**Figure 6:**
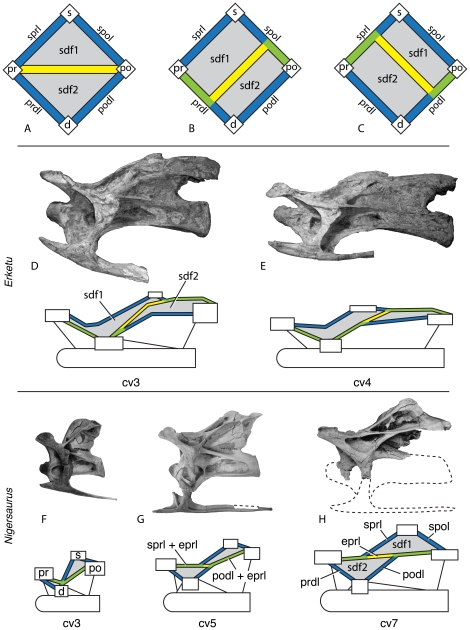
Variable development of the epipophyseal-prezygapophyseal lamina (eprl) and the divided spinodiapophyseal fossa (sdf) in cervical vertebrae. The eprl in its most basic form (**A**) connects the prezygapophysis directly with the epipophysis of the postzygapophysis, dividing the sdf into upper (sdf1) and lower (sdf2) subfossae. More commonly, the eprl is conjoined for at least a portion of its length with two or more laminae (**B**, **C**), although sdf1 and sdf2 are still readily identifiable. Blue (sprl, spol, prdl, podl) and yellow (eprl) bars represent single laminae; green bars represent conjoined laminae. Examples of conjoined eprl and the indentification of the fossae they bound are given using the holotypic cervical vertebrae of *Erketu ellisoni* (IGM 100/1803; **D**, **E**) and *Nigersaurus taqueti* (MNN-GAD 512; **F–H**) in left lateral view, with diagrammatic representation of laminae and landmarks bounding the sdf. Development of the eprl dividing the sdf is dependent on relative position of landmarks, particularly the separation of the summit of the neural spine (s) and the postzygapophysis (po), as well as the relative positions of the prezygapophysis (pr) and diapophysis (d). Even in taxa with a strongly developed eprl, such as *Nigersaurus*, the lamina is separate from either the sprl or the podl for only a short distance. Taxa with extremely elongate cervical vertebrae, such as *Erketu*, may have a slightly different arrangement of connectivity between the eprl, spino-zygapophyseal laminae, and zygapophyseal-diapophyseal laminae, although the presence of the eprl can still be traced. Seventh cervical vertebra of *Nigersaurus* reversed from right lateral. Not to scale.

In many neosauropods, some non-neosauropods (e.g., *Mamenchisaurus*), and theropods (e.g., abelisauroids), the spinodiapophyseal fossa (sdf) of cervical vertebrae is divided into two smaller fossae by the epipophyseal-prezygapophyseal lamina (eprl), which connects the epipophysis and prezygapophysis. The divided sdf constitutes a problematic arrangement of landmarks in the system outlined above: one of the resultant fossae is defined by all three primary landmarks (diapophysis, prezygapophysis, postzygapophysis) but no secondary landmark, because it is separated from the neural spine by the eprl; the other resultant fossa is defined by two non-adjacent primary landmarks (prezygapophysis, postzygapophysis) and a secondary landmark (neural spine). Because these two fossae constitute cases outside the naming system we have described, and because no convenient locational shorthand exists (as it does for fossae in divided laminae), we refer to them as sdf1 and sdf2, respectively (Figs. 4, 6). Sdf1 is bounded by the neural spine and zygapophyses, and sdf2 is bounded by the diapophyses and zygapophyses. In the simplest cases (e.g., *Afrovenator*; MNN TIG1-19), sdf1 is dorsal to the eprl, and sdf2 is ventral to the eprl. However, this orientation is altered in more complex cases, in which cervical vertebrae are elongate and the eprl combines with adjacent laminae; in some cases (e.g., Fig. 6F,G) the eprl is conjoined with other laminae for almost its entire length.

Identification of the eprl, and thus of sdf1 and sdf2, is made difficult by the variable development of the eprl in certain taxa. The eprl can occur as a low ridge (e.g., *Camarasaurus*), as a sharply demarcated lamina (e.g., *Nigersaurus* and *Euhelopus*), or as a lamina whose ends are conjoined with adjacent laminae (e.g., *Nigersaurus* and *Erketu*). This last case is perhaps the most confusing, because the eprl appears as a short strut that contacts other laminae throughout the cervical series. This typically occurs in elongate vertebrae, in which the configuration of the eprl is determined by the relative positions of the landmarks that define the spinal laminae (i.e., neural spine, pre- and postzygapophyses, diapophysis). For example, in the cervical vertebrae of *Nigersaurus*, the eprl is conjoined with the sprl anteriorly and podl posteriorly (Fig. 6F–H). In these anteroposteriorly elongate vertebrae, the neural spine and postzygapophysis are well separated from the prezygapophysis and diapophysis. This results in the sprl becoming oriented almost parallel to the podl and a very short ‘free’ portion of eprl that is not conjoined with adjacent laminae (Fig. 6). The length and orientation of the “free” portion of the eprl varies depending on the position of the relevant landmarks (compare cv 5 and cv 7 in Fig. 6). Although in most taxa the eprl closely parallels or merges with the sprl and/or the podl, in some taxa with extremely elongate cervical vertebrae (e.g., *Erketu*) the eprl instead contacts the prdl, the podl, and spol (Fig. 6). We refer to these fossae with the same name (sdf1, sdf2) in both *Erketu* and *Nigersaurus* because we interpret them as being defined by identical landmarks that have been altered by slightly different connections between the eprl and spino-zygapophyseal and zygapophyseal-diapophyseal laminae.

#### Fossae within divided laminae

Vertebral laminae are occasionally split into paired rami that retain the connections of the original lamina [9]. For example, the cprl is split in diplodocoids and *Mamenchisaurus*, the cpol is split in some vertebrae of *Camarasaurus* ([12]: pls. 70–73), the spol is split in *Barapasaurus* and more derived sauropods [7], the spdl is divided in *Epachthosaurus* and some other titanosaurs [28], and the pcdl is split in some vertebrae of *Saltasaurus* (J. A. Wilson and M. D. D'Emic pers. obs.). The fossae present between the rami of the divided laminae can be accommodated in our system by adding “fossa” to the end of the name of the lamina, or an “-f” to the end of the abbreviation for that lamina. For example, the fossa within a divided cprl would be the centroprezygapophyseal lamina fossa, or cprl-f (Fig. 4). We refrain from naming fossae within divided laminae using the two landmarks the divided lamina connects, because that would create a name redundant with one applied to a different fossa.

### Advantages of the Proposed Nomenclatural System

Although there are limitations to the system we propose, in our view these are outweighed by its simplicity, intuitiveness, and scalability. The names produced are far simpler than compound words formed by two or three laminae, and they can easily be simplified into short, easily decoded abbreviations. Because the proposed system for naming fossae parallels the one developed for vertebral laminae a decade ago [9], we anticipate that names created for fossae will be intuitive. Anyone who has achieved some fluency in vertebral laminae will be able to translate and produce names for neural arch fossae because the nomenclature uses the same landmarks. A spinodiapophyseal fossa and spinodiapophyseal lamina are both located on the same area of the neural arch.

Additionally, the system is scalable in the sense that it can be used to create names for new fossae by adding new landmarks. It is also scalable in the taxonomic sense, because these terms can be applied to non-saurischian taxa that have vertebral laminae that define fossae (e.g., crocodile-line archosaurs, snakes, temnospondyls). Because these terms are strictly descriptive, they can be applied to suitable anatomy without specific knowledge of the origin or function of that feature [27].

The sacral region presents a difficult case. Because many landmarks are obliterated or coalesced by fusion in skeletally mature individuals (e.g., zygapophyses), application of tripartite names is inadvisable. While we recognize the presence of laminae and fossae in the neural arches of sacral vertebrae, we refrain from applying tripartite names to them. Instead, we recommend applying more general, bipartite names if required (e.g., “centrodiapophyseal fossae”).

## Discussion

### Vertebral fossae in basal sauropods, macronarians, and diplodocoids

Below we provide examples demonstrating how nomenclature for vertebral fossae can be applied to a broad sampling of sauropod dinosaurs.

#### Basal sauropods (Figs. 7, 8)

*Tazoudasaurus naimi* is a basal sauropod from Morocco [29], [30]. With several well-preserved vertebrae, it is a good exemplar for the pattern of neural arch fossae in basal sauropods. Fossae are present on the axis, in particular near the podl. A shallow sdf is present on the lateral neural spine, and a relatively large pocdf lies ventral to it. These fossae are prominent in postaxial cervical vertebrae (CSPGM To1-354), which also have deeply invaginated fossae associated with the prezygapophyses (cprf, sprf; [29]: fig. 9; J. A. Wilson pers. obs.).

**Figure 7:**
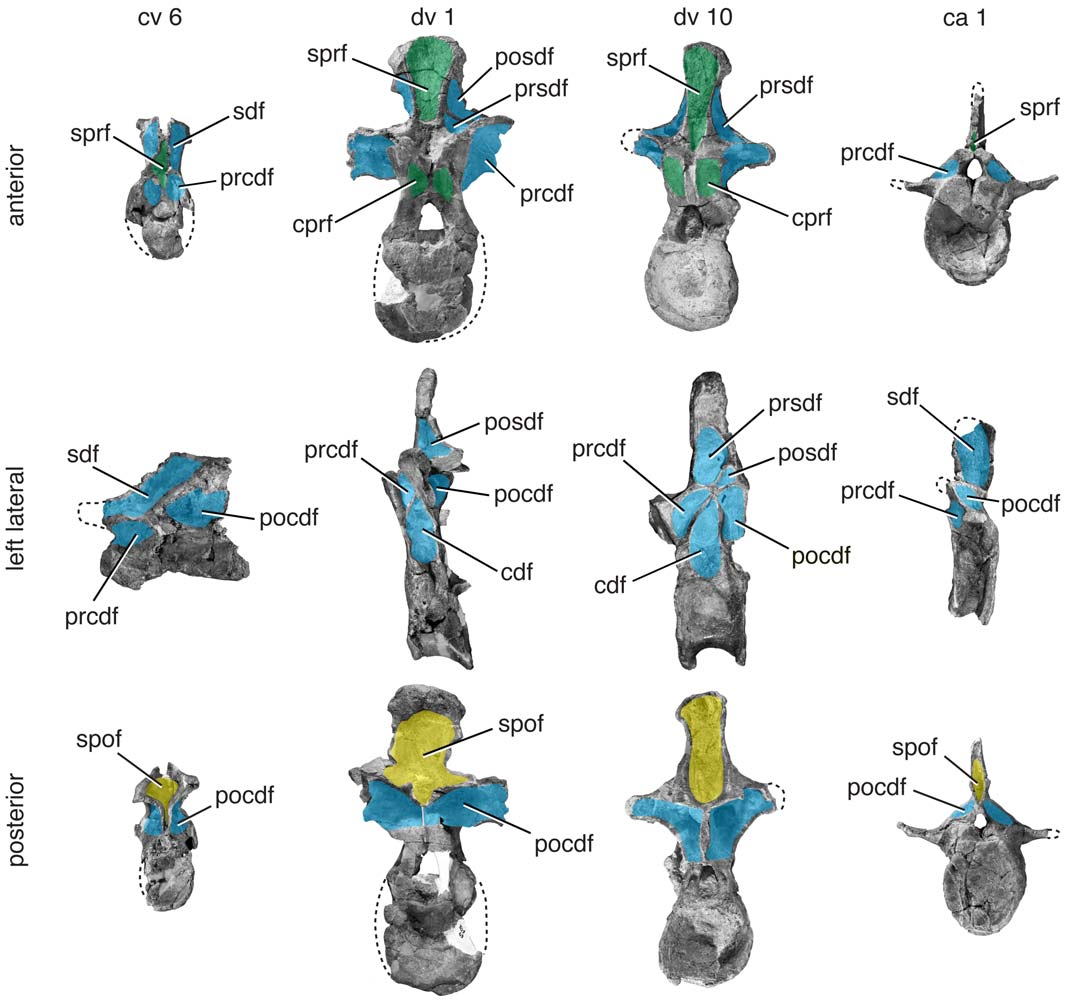
Representative vertebrae of *Tazoudasaurus naimi*. Anterior (top), left lateral (middle), and posterior (bottom) views of mid-cervical (ca. cv 6), anterior dorsal (ca. dv 1), posterior dorsal (ca. dv 10), and anterior caudal (ca. ca 1) vertebrae. Specimens come from several individuals referred to *Tazoudasaurus* and are scaled relative to one another. Mid-cervical vertebra CSPGM To1-354 is reversed from the original right lateral view. Important changes include reduction of the size of the cdf along the vertebral column. Diapophyseal fossae are in blue, prezygapophyseal fossae are in green, postzygapophyseal fossae are in yellow, and parapophyseal fossae are in orange. Images are modified from [29]:figs. 9, 11, 14–16). Abbreviations, **ca**, caudal vertebra; **cdf**, centrodiapophyseal fossa; **cpol-f**, centropostzygapophyseal lamina fossa; **cpaf**, centroparapophyseal fossa; **cpof**, centropostzygapophyseal fossa; **cprf**, centroprezygapophyseal fossa; **cprl-f**, centroprezygapophyseal lamina fossa; **cv**, cervical vertebra; **dv**, dorsal vertebra; **pa**, parapophysis; **pacdf**, parapophyseal centrodiapophyseal fossa; **pacprf**, parapophyseal centroprezygapophyseal fossa; **pocdf**, postzygapophyseal centrodiapophyseal fossa; **posdf**, postzygapophyseal spinodiapophyseal fossa; **prcdf**, prezygapophyseal centrodiapophyseal fossa; **prcpaf**, prezygapophyseal centroparapophyseal fossa; **prpadf**, prezygapophyseal paradiapophyseal fossa; **prsdf**, prezygapophyseal spinodiapophyseal fossa; **sdf**, spinodiapophyseal fossa; **spdl**, spindodiapophyseal lamina; **spof**, spinopostzygapophseal fossa; **sprf**, spinoprezygapophseal fossa; **sprl**, spinoprezygapophyseal lamina; **spol-f**, spinopostzygapophyseal lamina fossa.

**Figure 8:**
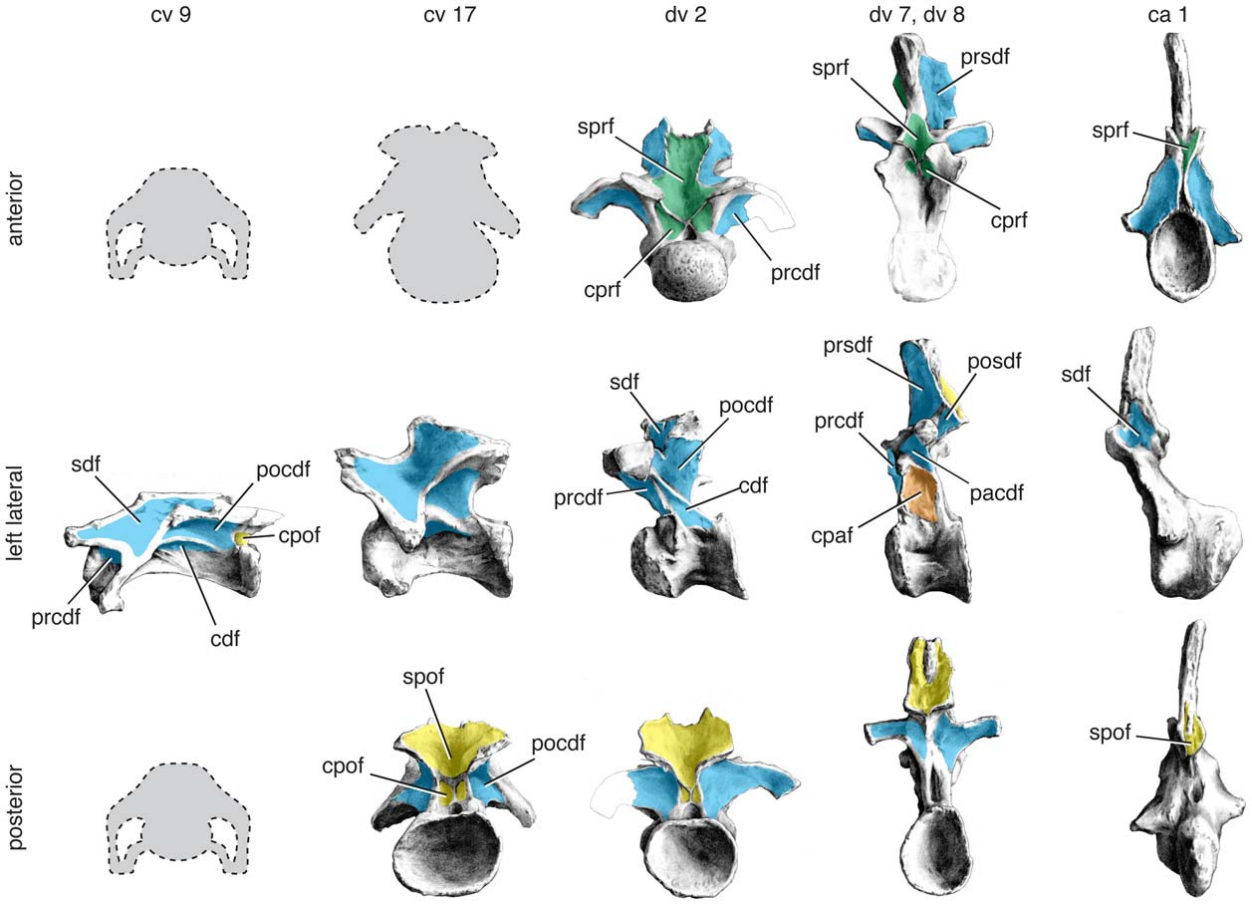
Representative vertebrae of *Mamenchisaurus youngi*. Anterior (top), left lateral (middle), and posterior (bottom) views of anterior cervical (cv 9), posterior cervical (cv 17), anterior dorsal (dv 2), posterior dorsal (dv 8), and anterior caudal (ca 1) vertebrae. Specimens come from a single individual (holotype ZDM 0083) and are to scale. Posterior view of cv 9 and anterior views of cv 9, cv 17, and dv 8 not available; anterior view of dv 7 used for dv 8. Important changes along the column include the appearance of a pcpl and the division of the cdf into a pacdf and a cpaf. Images are modified from [33]:figs. 15, 17, 20, 26, 30). Abbreviations and color scheme as in Figure 7.

In dorsal vertebrae, the appearance of parapophyseal and spinodiapophyseal laminae alters the arrangement of neural arch fossae. When the spdl first appears, it does not extend all the way to the neural spine by itself. Rather, it contacts the sprl near its base, creating a small, rounded prsdf (CSPGM To-1-69; J. A. Wilson pers. obs.). In this same vertebra, the parapophysis is still on the centrum, and the arrangement of centrodiapophyseal fossae in the cervical region is retained. In middle to posterior dorsal vertebrae (e.g., CSPGM To1-156), the parapophysis is low on the neural arch, and the spdl is more prominent. The parapophysis is associated with the a prpadf, but no additional laminae (e.g., prpl, acpl, pcpl) are present to create additional fossae. In contrast to anterior dorsal vertebrae, here the spdl contracts the spol instead of the sprl. In these vertebrae, the prsdf is large and the posdf is shallow and small.

The anterior caudal vertebrae are simple in design compared to the presacral vertebrae. The sprf in particular is narrow and deep, and the sdf is no longer divided. The prcdf, pocdf, and cdf are shallow, scooped-out hollows. As lamination diminishes in more posterior caudal vertebrae, these fossae disappear.

*Mamenchisaurus* is a non-neosauropod eusauropod based on several nearly complete skeletons [31]–[33]. *Mamenchisaurus*, along with its sister taxon *Omeisaurus*, has been resolved as a derived eusauropod just outside Neosauropoda [34]. The description of *Mamenchisaurus* vertebrae here is based on *Mamenchisaurus youngi* [33] and *M. hochuanensis* [32], both of which are known from nearly complete, articulated vertebral columns lacking only posterior caudal vertebrae.

The cervical vertebrae of *Mamenchisaurus* are elongate but dorsoventrally low, which warps the shape of the vertebral laminae and fossae. A shallow sdf is present on the lateral aspect of the neural spine. In some middle cervical vertebrae of *M. youngi*, a subtle horizontal ridge subdivides the sdf ([33]:fig. 15E, H). This may represent an incipient epipophyseal-prezygapophyseal lamina (see “The eprl and the spinodiapophyseal fossa”). A low, elongate pocdf is also present in anterior cervical vertebrae. In more posterior cervical vertebrae, which are taller and less elongate, the sdf and pocdf are accordingly modified in shape. The sdf becomes deeper in more posterior cervical vertebrae. A small prcdf appears to be present throughout the cervical series but is obscured in lateral view by the diapophysis.

The anteriormost dorsal vertebrae of *Mamenchisaurus* have a configuration of fossae somewhat similar to that of the posterior cervical vertebrae, due to the position of the parapophysis on the centrum. In these vertebrae, a small spdl is present and appears to contact the sprl, as described above for *Tazoudasaurus* ([33]:pl 8, fig. 5; [32]:fig. 6). The sdf is divided into a small prsdf and relatively large posdf. In more posterior dorsal vertebrae, the condition is reversed, and the spdl contacts the podl to form a large prsdf and a relatively small posdf. In the anterior dorsal vertebrae of *M. hochuanensis*, both the prdl and cprl are divided and enclose deep prdl-f and cprl-f, respectively, features that may be diagnostic of the species or genus [34]. By dv 4, the parapophysis is completely on the neural arch. In addition to the ppdl and acpl, the parapophysis bears a small pcpl that joins the pcdl before reaching the centrum. As such, it divides the cdf into a large cpaf and a much smaller, triangular pacdf. As the parapophysis migrates higher in the posterior dorsal vertebrae, the pcpl is reduced and eventually disappears, leaving only a large cpaf is confluent with the pleurocoel. A deep sprf, spof, cprf, and cpof are present, although they become shallower and less pronounced in more posterior dorsal vertebrae.

The caudal vertebrae show some lamination that define a small rounded sdf, a very narrow cprf, an sprf, and a shallow prcdf. Laminae and fossae are reduced or absent in middle and posterior caudal vertebrae.

#### *Camarasaurus* (Fig. 9)

The Upper Jurassic sauropod *Camarasaurus* is the most common sauropod from North America [35], [36] and considered to be one of the basalmost macronarians [7]. *Camarasaurus* (Gr. “chambered lizard”) was originally named for the pair of large, deep hollows in the cervical and dorsal centra [37]. In addition to pneumatic centra, the presacral vertebrae also exhibit large neural arch fossae.

**Figure 9:**
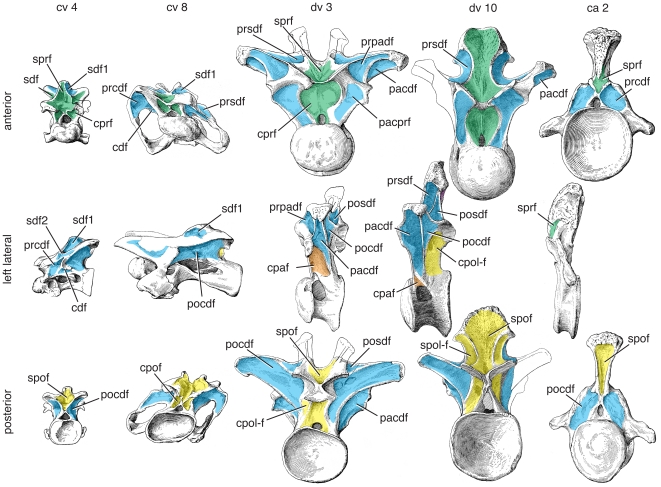
Representative vertebrae of *Camarasaurus supremus*. Anterior (top), left lateral (middle), and posterior (bottom) views of anterior cervical (ca. cv 4), mid-cervical (ca. cv 8), anterior dorsal (ca. dv 3), posterior dorsal (ca. dv 10), and anterior caudal (ca. ca 2) vertebrae representing multiple individuals (AMNH 5761/X-3; AMNH 5761/X-7; AMNH 5761-a/D-X-106); AMNH 5760′/D-X-125; AMNH 5761/Cd-X-2) and are to scale. Positions of vertebrae were assigned based on comparisons with complete axial series. Important changes along the column include division of the spinodiapophyseal fossa (sdf) is divided into two smaller fossae (sdf1, sdf2) by the epipophyseal prezygapophyseal lamina (eprl) in anterior and middle cervical vertebrae. The eprl is more subtly developed or absent in more posterior cervical vertebrae (see text for discussion). Images are modified from [12] (pl. 67, figs. 4, 8; pl. 70, fig. 10; pl. 71, fig. 2). Abbreviations and color scheme as in Figure 7.

Cervical vertebrae of *Camarasaurus* usually exhibit two main fossae in lateral view, the sdf and pocdf (e.g., [12]:pl. 67–69). The pocdf is the most conspicuous fossa in the cervical series, forming a deep, triangular fossa in more anterior cervical vertebrae, and a more elongate triangular fossa in more posterior cervical vertebrae. Although anterior cervical vertebrae exhibit a single large pocdf, it is subdivided by irregular laminae into smaller fossae in posterior cervical vertebrae (e.g., *C. supremus*; AMNH 5760, 5761). The sdf is present on the lateral aspect of the neural arch, bounded anterodorsally by the sprl and posteroventrally by the pcdl. In *Camarasaurus*, these two laminae approach each other near midlength, occasionally linked by a short, horizontal lamina that probably represents an incipient eprl ([12]:pl 67; CM 11338, J. A. Wilson pers. obs.). This short, somewhat intermittent eprl variably subdivides the sdf into sdf1 and sdf2, as in some titanosauriform (e.g., *Euhelopus* [20]) and rebbachisaurid (e.g., *Nigersaurus* [38]) sauropods, as well as in abelisaurid theropods (e.g., *Majungasaurus* [39]). Whether single or divided by a short eprl, the sdf is shallow in cv 3–5 but more deeply hollowed in more posterior cervical vertebrae. Below the diapophysis, the cdf extends from the anterior to the posterior margin of the neurocentral junction. The boundary between the cdf and the pleurocoel can be very weak or absent. A small but well defined prcdf is present throughout the cervical series, but it is obscured by the diapophysis and cervical rib in all but the most posterior cervical vertebrae. The cprf and cpof are present in all postaxial cervical vertebrae. The cprf tends to be wider than the cpof, due to the wider separation of the right and left cprl compared to the right and left cpol. The sprf occupies nearly the entire anterior surface of the neural spine in cv 3–5/6.

In anterior dorsal vertebrae, the parapophysis is situated on the centrum or at the base of the neural arch, and the configuration of centrodiapophyseal fossae resembles that in the cervical series. As it rises onto the neural arch, the parapophysis contacts the diapophysis via the ppdl, the parapophysis via the prpl, and the centrum via the acpl. Together with the prpl, these laminae bound a small prpadf. The parapophysis develops a weak pcpl, which subdivides the fossa beneath the diapophysis into a pacdf and cpaf (see Fig. 5). In more posterior dorsal vertebrae, the pcdl is oriented subvertically and positioned midway between the prezygapophyses and postzygapophyses. As a consequence, the pcdl no longer contacts the posterior aspect of the centrum. The fossa posterior to the pcdl is divided by the lateral portion of the cpol. As a consequence, the pocdf is reduced in size and a fairly large fossa (i.e., the cpol-f) that separates the medial and lateral portions of the cpol is conspicuous in lateral view. In the anteriormost dorsal vertebrae, the spdl is relatively weakly developed and contacts the sprl near its base to form a small prsdf ([12]:pl. 73, fig. 1), as noted for *Tazoudasaurus* above. More posteriorly, the spdl contacts the podl near the neural spine to form a tall, triangular posdf. In these middle dorsal vertebrae, the neural arch pedicles are elongated, creating an elongate cprf, especially in *Camarasaurus grandis* [40]. Posterior dorsal vertebrae bear a divided spol, the medial and lateral branches of which define an intralaminar fossa, the spol fossa (spol-f).

The spol is well-developed in ca 15–20 of *Camarasaurus*, defining an spof on the posterior surface of the neural spines. Ca 1 and 2 have a deep spof that occupies much of the posterior surface of the neural spine. The depth of the spof rapidly decreases across ca 3–15, and by ca 16–25, this fossa is restricted to the base of the spine. A well-developed acdl is present in ca 1 and 2, partially bounding a shallow cprf that is visible anteriorly. The deep transverse process partially bounds a posdf and pocdf, which is visible posteriorly. Lamination (and thus the development of fossae) is greatly reduced in more posterior caudal vertebrae.

#### *Brachiosaurus* (Fig. 10)

The Late Jurassic species *Brachiosaurus brancai* and *Brachiosaurus altithorax*, which are considered by some to be separated at the subgeneric [41] or generic [42] level, are the basalmost members of Titanosauriformes, a clade of sauropods that originated in the Middle Jurassic and diversified during the Cretaceous [7]. Titanosauriformes and its constituent subclades evolved dramatic changes in their vertebral morphology, including reclined neural spines (e.g., *Rinconsaurus* [43]), strongly pointed epipophyses and pre-epipophyses (e.g., *Phuwiangosaurus* [44]), and camellate (“spongy”) pneumatic internal bone structure [7]. Because *Brachiosaurus* is well-preserved and a basal member of this morphologically diverse clade, it is chosen as an exemplar here.

**Figure 10:**
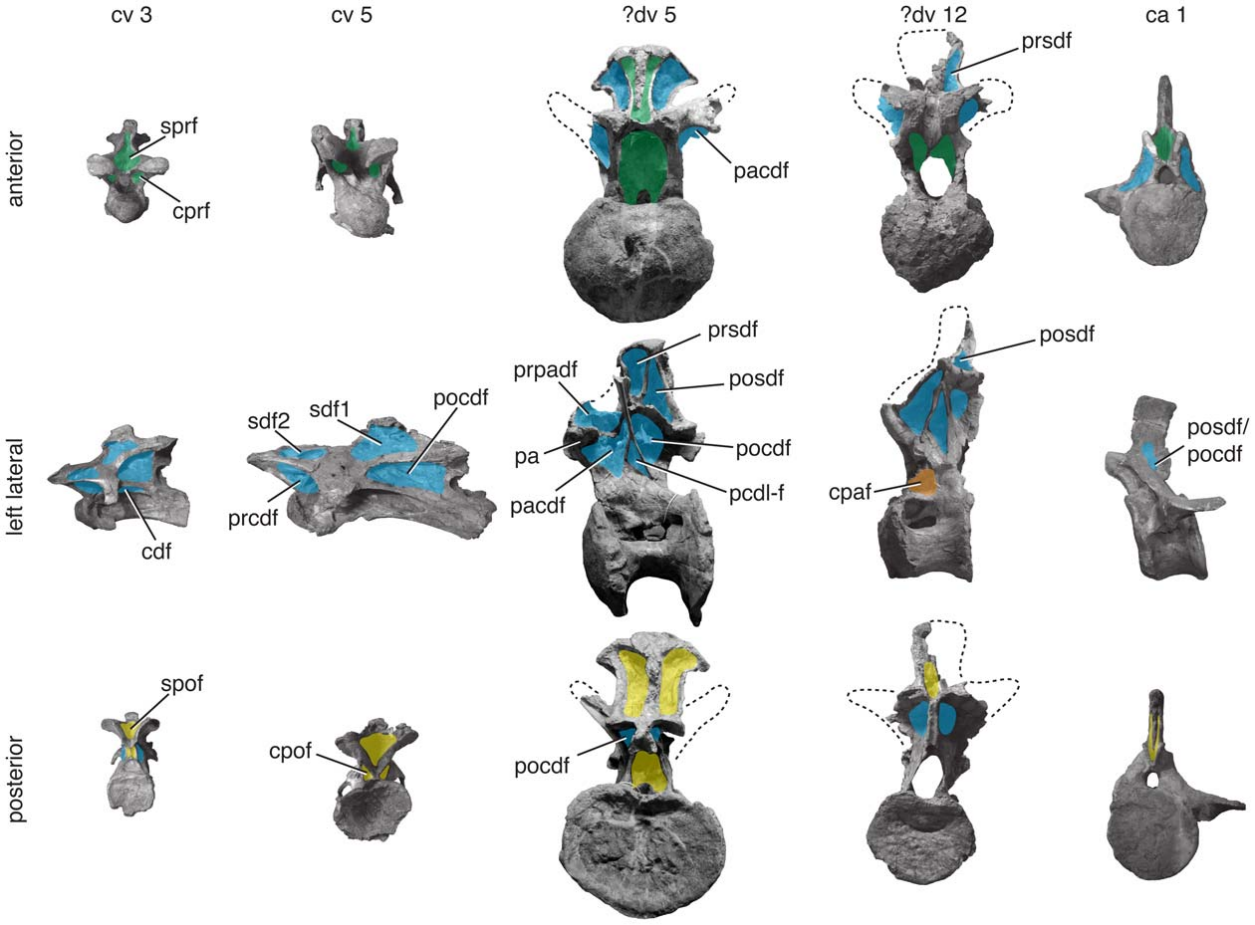
Representative vertebrae of *Brachiosaurus brancai*. Anterior (top), left lateral (middle), and posterior (bottom) views of anterior cervical (cv 3), middle cervical (cv 5), anterior dorsal (?dv 5), posterior dorsal (?dv 12), and anterior caudal (ca 1) vertebrae. Specimens come from several skeletally mature individuals (MB.R. 2180, MB.R. 3824, MB.R. 3822, MB.R. no number, see [45]: pl. 2) and are to scale. Important changes along the column include the appearance of numerous irregular fossae in the sdf of cervical vertebrae and the absence of a cpaf in mid-dorsal vertebrae. Green/blue gradient in the lateral view of ca 1 indicates an undistinguishable pocdf + posdf. Photographs of ?dv 12 and ca 1 have been reversed. Abbreviations and color scheme as in Figure 7.

The cervical vertebrae of *Brachiosaurus brancai* have deep, well-defined fossae. On the lateral aspect of the neural spine, the sdf contains many smaller fossae that are irregularly distributed and bears a crenulated, polished texture, as in other brachiosaurids (e.g., *Sauroposeidon* [23]). Below the zygodiapophyseal table, the pocdf, prcdf, and cdf occasionally contain smaller, irregular fossae resembling those in the sdf. Deep, single sprf, spof, cprf, and sprf are also present. In some cervical vertebrae, fossae are present within subdivided laminae (e.g., cpol-f in cv 7; [45]:fig. 31).

Posterior cervical and anteriormost dorsal neural arches are missing in *Brachiosaurus brancai*, but they are present in dv ?3 and ?4, the latter being more complete. The migration of the parapophysis onto the neural arch in these dorsal vertebrae divides the anterior centrodiapophyseal lamina (acdl) of the cervical vertebrae into the acpl and ppdl. The acpl and pcpl bound the cpaf dorsally, and the latter lamina forms the anteroventral border for the tall, subdivided pacdf. A fourth lamina, the prpl, joins the parapophysis to the prezygapophysis and bounds the prpadf and pacprf.

In dorsal vertebrae, the space occupied by the sdf in cervical vertebrae is bisected by the spdl to form a prsdf and posdf. Anteriorly and posteriorly, the sprf and spof are deep near the base of the spine but weak towards its apex, as the sprls and spols converge, and the prsl and posl become more prominent.

Caudal vertebrae of *Brachiosaurus brancai* (MB.R. 2921) and *Brachiosaurus altithorax* (FMNH P 25107) have fewer neural arch laminae than do presacral vertebrae. A prominent fossa is present on the anterior aspect of the transverse process in the first few caudal vertebrae. The fossa is bounded anteriorly by the cprl and posteriorly by the transverse process and the laminae that emanate from it, which we interpret to be the acdl and prdl. Following this interpretation, this fossa is bounded by the prezygapophysis, diapophysis, and centrum and can be identified as the prcdf. A fossa is present just in front of the postzygapophyses that represents one or both the posdf and pocdf.

#### *Rapetosaurus* (Fig. 11)

Titanosaurs are recognized as one of the major radiations of sauropods [46], and their axial anatomy is becoming better understood with the discovery of nearly complete vertebral columns [21], [22], [47]. These discoveries have revealed morphological disparity in the axial skeleton of the clade, including differences in the height, proportions, and anteroposterior inclinations of the neural spines of cervical and dorsal vertebrae. Neural arch laminae and fossae can be poorly developed in some titanosaur vertebrae (e.g., cervical vertebrae of *Malawisaurus* [48]) or so well-developed that fossae reach the midline (e.g., posterior cervical/anterior dorsal vertebrae of *Mendozasaurus* [21]). *Rapetosaurus krausei* from the Late Cretaceous of Madagascar is chosen as an exemplar here because of its completeness and importance to titanosaur phylogeny [34], [49]. *Rapetosaurus* also presents an excellent case for reconstructing the pneumatic anatomy of a titanosaur, because one of the signature osteological correlates of bony pneumaticity, crenulated and polished texture [3], is well preserved [22].

**Figure 11:**
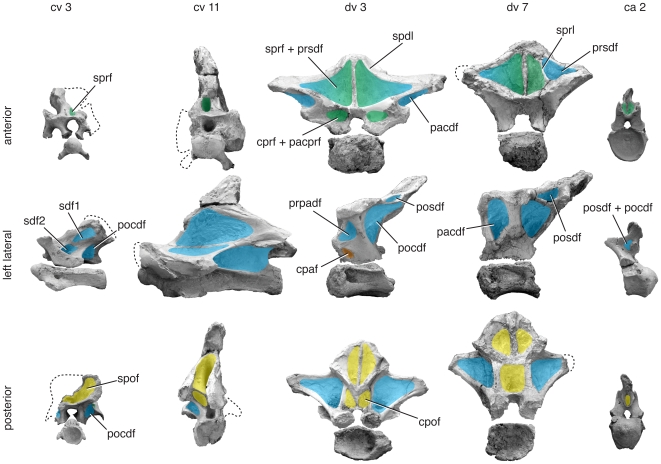
Representative vertebrae of *Rapetosaurus krausei*. Anterior (top), left lateral (middle), and posterior (bottom) views of anterior cervical (cv 3), posterior cervical (cv 11), anterior dorsal (dv 3), posterior dorsal (dv 7), and anterior caudal (ca 2) vertebrae. Specimens represent a single juvenile individual (FMNH PR 2209) and are to scale. Several important changes along the column are related to the anteroposteriorly shortened and reclined neural spines of the pectoral region, including the development of broad, flat sprfs, loss and re-emergence of the sdf/posdf. As in many other titanosaurs, there is a broad, subtly divided sdf that bears crenulated texture in the mid-cervical region and a dorsally restricted pacdf in the mid-dorsal region. Green/blue gradient in the lateral view of ca 2 indicates that the pocdf and posdf cannot be distinguished from one another. Images modified from [22]; photographs of dv 3 and dv 7 have been reversed. © Copyright 2009 The Society of Vertebrate Paleontology. Reprinted and distributed with permission of the Society of Vertebrate Paleontology. Abbreviations and color scheme as in Figure 7.

The cervical vertebrae of *Rapetosaurus* have laminae and fossae similar to other neosauropods described here. The cervical vertebrae possess an eprl that is best developed in cv 3 and less well-developed in cv 4 and cv 9 (intervening vertebrae are not well preserved [22]). The eprl subdivides the sdf into two separate fossae, sdf1 and sdf2. By the eleventh cervical vertebra, the eprl is absent or represented by a very subtle ridge incompletely dividing the sdf ([22]:fig. 10), as in some other titanosauriforms (e.g., *Euhelopus* cv 14; [20]). Fossae below the zygapophyses, the cprf and cpof, are either absent (e.g., cv 3) or poorly defined (e.g., cv 9) in cervical vertebrae. The sprf and spof are pit-like and nearly meet within the neural spine of anterior cervical vertebrae. They are shallower and broader as the neural spine changes shape more posteriorly along the axial column.

Posterior cervical and anterior dorsal vertebrae, informally termed “pectoral” vertebrae, are morphological intermediates between the cervical vertebrae they follow and the dorsal vertebrae they precede. In pectoral vertebrae, the neural spine becomes anteroposteriorly short, and the sprl does not actually reach the prezygapophysis. In the 17th presacral vertebra, which is either the last cervical or the first dorsal vertebra, the sprl terminates at the base of the neural spine ([22]:fig. 13f). No well developed lamina extends to the prezygapophysis or diapophysis, but there is a subtle ridge that extends in the direction of the former. This ridge is flanked laterally by a pneumatic foramen, better developed on the left than on the right side, that may represent a very reduced spinodiapophyseal fossa (sdf1). In this transitional vertebra, it is difficult to determine the identity of the structure separating it from the larger spinodiapophyseal fossa on the lateral aspect of the neural spine, which could be either the eprl or the spdl. In this same vertebra, a similar pneumatic foramen (again, better developed on the left than on the right side) is found at the base of a thick diapophyseal lamina. We interpret this pneumatic foramen to be a reduced cdf that incipiently separates acdl and pcdl; this identification is confirmed in more posterior vertebrae ([22]:fig. 13e, 15c). In the 18th presacral vertebra (either dv 1 or 2), the sdf is reduced or absent because diapophyseal laminae (podl, spdl) and spinal laminae (sprl) are indistinguishable; a single lamina is visible in lateral view ([22]: fig. 14c). By the 19th presacral vertebra (dv 2 or 3), these laminae have begun to separate from one another, and a subtle sprl is visible anteriorly, and a prsdf is visible between it and the spdl. A small posdf opens between the spol and spdl, which is difficult to differentiate from the podl ([22]:fig. 15). By the 20th presacral vertebra (dv 3 or 4), the podl and spdl are more easily identifiable as separate laminae, and a small posdf is visible laterally ([22]:fig. 16c). The posdf increases in size and depth as the neural spine becomes more upright towards the sacrum. In anterior and middle dorsal vertebrae, the neural spine is anteroposteriorly compressed, and the sprl is reduced or absent. The large fossae visible in anterior view, which are subdivided by the prsl, represent both the prsdf and the sprf, either separately when the sprl is present, or combined when it is absent. In the 20th presacral vertebra (dv 3 or 4), the parapophysis is positioned on the neural arch and bounds the pacdf above and behind it, cpaf below it, and combined prpadf above and in front of it. In dv 3 and more posterior dorsal vertebrae, the cprl is reduced or absent, as in *Neuquensaurus* [34], resulting in a confluent pacprf and cprf. The pacdf persists throughout the dorsal vertebrae as a sharply defined fossa between the diapophysis and parapophysis. This fossa does not extend ventrally to the neurocentral junction, resembling the condition in some other titanosaurs (e.g., *Trigonosaurus* [50]). The cpaf has a more restricted presence, limited to dv 2–4. The pocdf is prominent throughout the dorsal vertebral column as in the cervical vertebrae and is sometimes subdivided (e.g., dv 4–5) by a vertical lamina, often only on one side. The cprf and cpof are absent or poorly defined in the dorsal vertebrae, with the exception of the last, which has a deep cprf ([22]:fig. 22).

Anterior caudal vertebrae of *Rapetosaurus* are poorly known, but two proximal caudal vertebrae bear a well-developed sprf and weak spof. A shallow fossa in front of the postzygapophysis may represent the posdf, pocdf, or combination of the two.

Interpreting the vertebral column of *Rapetosaurus* and other titanosaurs is complicated by the reduction or loss of the sdf as a consequence of coalescence of several laminae in the transition between cervical and dorsal vertebrae. Identification of vertebral laminae in these vertebrae can be challenging and influences the nomenclature applied to neural arch fossae. For example, an interpretation of the vertebral laminae of the titanosaur *Trigonosaurus* [51] differs from the one presented for morphologically similar fossae in *Rapetosaurus* above. As a consequence, the nomenclature applied to the neural arch fossae for each will differ between interpretations.

#### *Apatosaurus* (Fig. 12)

The vertebrae of *Apatosaurus* represent the general pattern of lamination seen in diplodocoid sauropods, but are not as complex as they are in some more derived taxa such as *Nigersaurus* (see below). The pattern of neural arch fossae in diplodocoids is affected by the presence of a divided lamina (a divided cprl in cervical vertebrae), a composite lamina (a lateral lamina formed by the sprl and spol in caudal vertebrae), and complex caudal lamination in the clade [9]. Most diplodocoid taxa (e.g., *Apatosaurus*, *Dicraeosaurus*) have bifurcated presacral neural spines, altering the expression of the sprf and spof.

**Figure 12:**
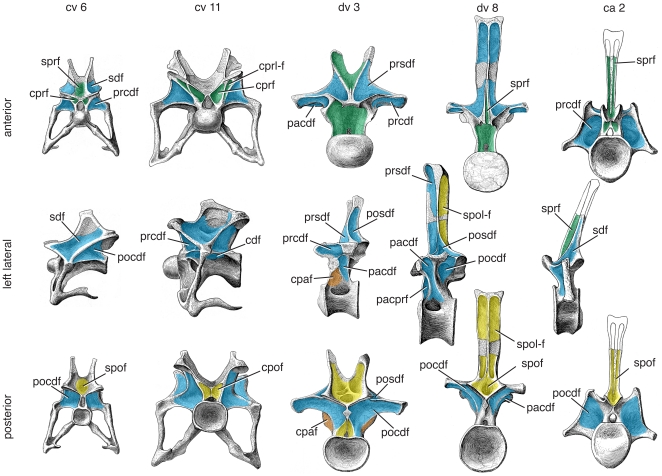
Representative vertebrae of *Apatosaurus louisae*. Anterior (top), left lateral (middle), and posterior (bottom) views of anterior cervical (cv 6), posterior cervical (cv 11), anterior dorsal (dv 3), posterior dorsal (dv 8), and anterior caudal (ca 2) vertebrae representing a single individual (CM 3018) and are to scale. Important changes along the column include the loss of the sprf and spof in bifid-spined posterior cervical and anterior dorsal vertebrae, appearance of the prcprf in posterior cervical vertebrae, and the division of the cdf into the cdf and cpaf in mid- and posterior dorsal vertebrae. Images modified from [52]:pls. 24–26). Abbreviations and color scheme as in Figure 7.

The cervical vertebrae of *Apatosaurus* have deep, well-defined diapophyseal fossae (sdf, cdf, prcdf, pocdf). The sdf is undivided and is deepest above the diapophysis. The deeply bifurcate neural spine limits the depth and size of the sprf and spof. In anterior cervical vertebrae, where bifurcation is absent (cv 1–3 or 4) or shallow (cv 4 or 5–7), the sprf and spof form deep pockets. In shallowly bifurcate anterior neural spines, these fossae are bounded anteriorly/posteriorly by the bone festooned between the metapophyses. This “webbing” more closely follows the margin of the divided spines in posterior vertebrae, and as a result the sprf and spof are reduced or absent. In some other diplodocoids (e.g., *Amargasaurus*; MACN N-15; J. A. Whitlock pers. obs.), this “webbing” is absent, and neither the sprf nor the spof can be identified. Mid- and posterior cervical vertebrae in diplodocids also bear an autapomorphic intralaminar fossa, the cprl fossa (cprl-f), formed in the space beneath the prezygapophyses and between the medial and lateral branches of the cprl. This fossa is more consistently present in diplodocines (e.g., *Diplodocus*, *Barosaurus*), but is at least intermittently present in *Apatosaurus* (e.g., cv 12 in CM 3018). A divided cpof is present in mid- and posterior cervical vertebrae, but not in the anteriormost vertebrae.

Dorsal vertebrae of *Apatosaurus* bear a spinodiapophyseal lamina, dividing the sdf vertically into a prsdf and posdf. In dv 1–2, the prsdf is visible only in anterior view; by dv 3 the spdl has shifted posteriorly relative to the sprl, and the prsdf can be seen in lateral view. The centrodiapophyseal laminae in the first two dorsal vertebrae are unaltered from their appearance in the cervical vertebrae, but by dv 3 the parapophysis has moved onto the neural arch and interrupts the acdl. A pcpl appears coincidently (albeit intermittently) with the dorsal shift of the parapophysis, creating a cpaf; where there is no pcpl, there is only the cdf. Rarely, the ppdl is divided (e.g., dv 6 in CM 3018; [52]), creating a subdivided fossa. The sprf and spof reappear in anterior dorsal vertebrae, as the metapophyseal “webbing” becomes more prominent and expansive dorsally. The neural spine is single by dv 4 or 5, and it bears a median lamina formed by conjoined sprl and the prsl. A small sprf is present between the sprl below their mutual contact. As a result, the prsdf is visible in anterior view. In the posteriormost dorsal vertebrae (e.g., dv 9 in CM 3018; [52]), the prsl and sprl are no longer conjoined, and the prsl divides the sprf. In posterior dorsal vertebrae, the spof is restricted dorsally by the conjoined medial spol, which forms the composite posl. In these vertebrae, an intralaminar fossa, the spol fossa (spol-f), appears between the medial and lateral branches of the divided spol. A cpof is present only in anterior dorsal vertebrae; mid- to posterior dorsal vertebrae do not have a well-defined concavity. The loss of the cpof is roughly concurrent with the first appearance of the hyposphene, although the features sometimes overlap (dv 3 in CM 3018; dv 4 in NSMT-PV 20375 [52], [53]).

As in other diplodocoids, anterior caudal vertebrae in *Apatosaurus* retain most of the lamination present in presacral vertebrae, including all four diapophyseal laminae [9]. An undivided sdf, prcdf, and pocdf are present. In caudal vertebrae, the sprl meet the spol to form a lateral lamina. Pre- and postspinal laminae divide the sprf and spof, which persist through the first 10–12 caudal vertebrae. The sprf faces anteriorly, giving the neural spine a similar appearance in both anterior caudal and posterior dorsal vertebrae. The cprf is present and divided in ca 4–5 and absent in more posterior vertebrae; the cpof is absent in all caudal vertebrae. The prcdf and pocdf disappear following the cprf.

#### *Nigersaurus* (Figs. 6,13)

*Nigersaurus* is a rebbachisaurid diplodocoid [54]. Although lightly built, its vertebral laminae and fossae largely conform to the pattern described for *Apatosaurus* (see above). The presence of novel laminae in the cervical vertebrae [38], [55] and the attendant alteration of the pattern of fossae necessitate further discussion, however.

**Figure 13:**
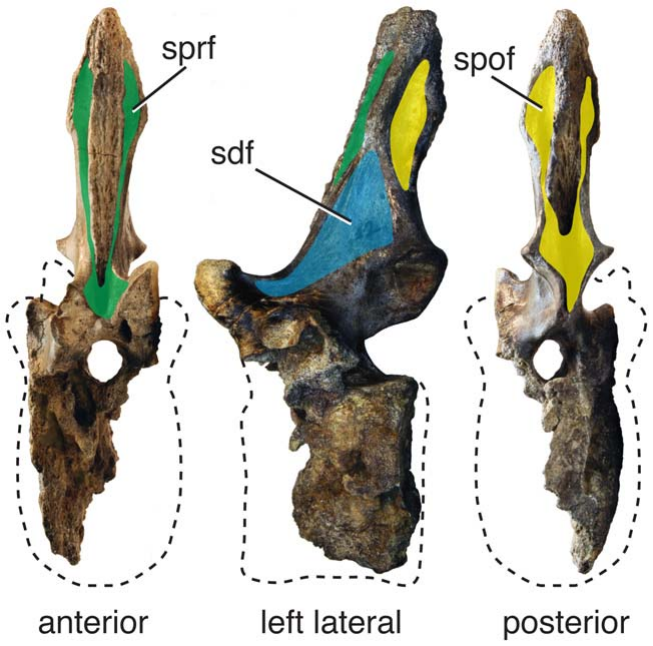
Anterior caudal vertebra of *Nigersaurus taqueti*. Anterior (left), left lateral (middle), and posterior (right) views of an anterior caudal vertebra (MNN GAD-516). Three fossae, the sprf, spof, and an undivided sdf, persist in anterior caudal vertebrae of *Nigersaurus*. The sprf and spof are divided distally by the prsf and posf, respectively. The presence or absence of centrodiapophyseal fossae cannot be determined. Abbreviations and color scheme as in Figure 7.

In cervical vertebrae of *Nigersaurus*, the sdf is divided by a roughly horizontal lamina, the epipophyseal-prezygapophyseal lamina (eprl), which connects the epipophysis and prezygapophysis. As in *Euhelopus* [20] and some titanosaurs (see above), the eprl subdivides the sdf horizontally. The two divisions of the sdf become sdf1 and sdf2. As in all non-flagellicaudatan diplodocoids, the neural spines of all vertebrae in *Nigersaurus* are undivided, but the laterally-oriented sprl results in an extremely shallow sprf. The spof is deep and diamond-shaped.

The dorsal vertebrae are similar in most regards to the unbifurcated vertebrae of *Apatosaurus*. As in other rebbachisaurids, however (e.g., *Rebbachisaurus*, MNHN 1957; *Limaysaurus*, MUCPv-205; J. A. Whitlock pers. obs.), the spol in dorsal vertebrae is reduced. It doesn't reach above one-third the height of the neural spine, where the spol merges with the the lateral lamina and/or the posl. As a consequence, the spof is greatly reduced in size, and the left and right spof are visible in lateral view. Although present and well developed in the last cervical vertebrae, the eprl is absent in dorsal vertebrae, which lack epipophyses.

Anterior caudal vertebrae in *Nigersaurus* retain much of the lamination present in presacral vertebrae, as in *Apatosaurus*. The sdf, sprf, and spof can be identified on the only known anterior caudal vertebra of *Nigersaurus*. Unfortunately, the transverse processes are too damaged to confidently identify any of the centrodiapophyseal fossae. The sprf is divided along its entire length by the prsl. In lateral view, the spof can be seen as a characteristically ovate depression in *Nigersaurus* [38] and other rebbachisaurids [55], similar to the condition in dorsal vertebrae.

### Neural arch fossae as phylogenetic data

The proposed system provides an unambiguous, landmark-based reference to neural arch fossae in sauropods and other reptiles, making these fossae readily accessible as character data for phylogenetic analysis. In all cases, characters developed for pneumatic fossae are dependent on the vertebral laminae that bound them, and thus coding both is redundant. However, although neural arch fossae are dependent on laminae, laminae are not dependent on fossae. For example, although many theropods have laminae associated with the neural spine and diapophysis (e.g., prdl, podl, sprl, spol, eprl), they rarely develop fossae dorsal to the zygodiapophyseal table. Likewise, several laminae are required to create most fossae, so referring to fossae rather than laminae will present different phylogenetic patterns than those of individual laminae, and may be more convenient in some cases. The proposed nomenclatural system can be used to code increases or decreases in the development of neural arch fossae in various clades on a precise, fossa-by-fossa basis. The fossae associated with the eprl may be considered as an example of novel phylogenetic characters associated with the nomenclatural system presented herein. Although the eprl itself has been mentioned in a character context for both theropods [56], [57] and sauropods [20], [38], the morphology of the fossae it bounds (i.e., sdf1, sdf2) has not. Variation in the shape and position of these fossae may prove a useful source character data in abelisauroids, diplodocoids, and titanosauriforms.

### Morphospace

**Figure 14:**
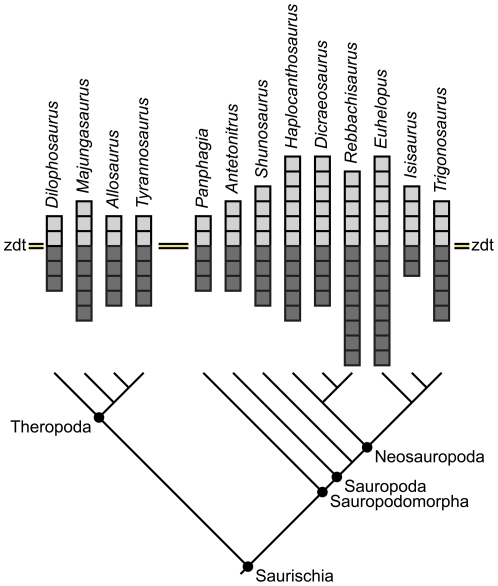
Neural arch fossa morphospace. Comparative diagram illustrating relative complexity of neural arch fossae in four theropods and nine sauropodomorphs. light gray squares represent unique fossae above the zygodiapophyseal table; dark gray squares represent unique fossae below the table. Unique fossae are defined as midline fossae and either the right or left paramedian fossae. Midline fossae that are subsequently divided count as two unique fossae. Therefore, a vertebra with two midline fossae (the sprf and spof) and two paramedian fossae (the prsdf and the posdf) is represented by four light gray squares. If a similar vertebra has a divided sprf but is otherwise identical, it is represented by five light gray squares. Note the general increase in complexity above and below the table in Neosauropoda compared to basal eusauropods (e.g., *Shunosaurus*), basal sauropods (e.g., *Antetonitrus*), basal sauropodomorphs (e.g., *Panphagia*), and theropods, although reduced complexity does occur in *Dicraeosaurus* and *Isisaurus*. Tree topology is based on several recent phylogenies [34], [46], [60], [61].

Application of a landmark-based nomenclature to neural arch fossae also facilitates broader, non-phylogenetic comparisons between taxa, including examination of patterns of morphospace occupation or of the degree of complexity of neural arch fossae. In a simple comparison of the complexity of neural arch fossae amongst several saurischians in which fossae are indicated by squares above and below the diapophyseal table (Fig. 14), note that in theropods and basal sauropodomorphs (i.e., “prosauropods”) possess fewer squares than derived sauropods. Derived sauropods (i.e., neosauropods) typically have more complex pneumaticity associated with the diapophysis and parapophysis below the zygodiapophyseal table, as well as pneumaticity associated with the diapophysis and neural spine above the zygodiapophyseal table. Some derived titanosauriforms (e.g., *Isisaurus*), however, appear to have reversed this trend towards complexity by reduction of neural arch lamination and external pneumaticity. The dicraeosaurid *Dicraeosaurus* also displays reduced fossa complexity; in *Dicraeosaurus* and other dicraeosaurids [55], this reduced complexity is coincident with reduced pneumaticity of the centrum. It should be noted, however, that these simplified comparisons illustrate only differences in complexity (i.e., the number of pneumatic spaces); inferences of the degree of pneumaticity present (e.g., [58]) and its relation to other pneumatic features will need to be based on further study.

### Conclusions

The nomenclatural system proposed here provides landmark-based names for vertebral fossae in sauropods and other saurischian dinosaurs. Simple combinations of primary, secondary, and tertiary landmarks allow us to create eight bipartite names and seven tripartite names that point to individual fossae (Fig. 3). In addition to these 15 names, many additional fossae represent special cases (i.e., fossae within divided laminae; subdivisions of the sdf; Fig. 4). The proposed nomenclatural system builds on conventions developed in previous studies of vertebral anatomy and is scalable both taxonomically and morphologically. It is designed to provide more precise and detailed descriptions of vertebral anatomy and to make those observations easily translatable into morphological characters that can be used in analyses of phylogeny and disparity.

## References

[1] Witmer LM (1997). The evolution of the antorbital cavity of archosaurs: a study in soft-tissue reconstruction in the fossil record with an analysis of the function of pneumaticity.. Society of Vertebrate Paleontology Memoir.

[2] Gauthier JA, Kluge AG, Rowe T (1988). Amniote phylogeny and the importance of fossils.. Cladistics.

[3] O'Connor PM (2006). Postcranial pneumaticity: An evaluation of soft-tissue influences on the postcranial skeleton and the reconstruction of pulmonary anatomy in archosaurs.. Journal of Morphology.

[4] Gower DJ (2001). Possible postcranial pneumaticity in the last common ancestor of birds and crocodilians: evidence from *Erythrosuchus* and other Mesozoic archosaurs.. Naturwissenschaften.

[5] Sereno PC, Martinez RN, Wilson JA, Varricchio DJ, Alcober OA (2008). Evidence for avian intrathoracic air sacs in a new predatory dinosaur from Argentina.. PLoS ONE.

[6] Claessens LPAM, O'Connor PM, Unwin DM (2009). Respiratory evolution facilitated the origin of pterosaur flight and aerial gigantism.. PLoS ONE.

[7] Wilson JA, Sereno PC (1998). Early evolution and higher-level phylogeny of sauropod dinosaurs.. Society of Vertebrate Paleontology Memoir.

[8] Wedel MJ (2009). Evidence for bird-like air sacs in saurischian dinosaurs.. Journal of Experimental Biology A.

[9] Wilson JA (1999). Vertebral laminae in sauropods and other saurischian dinosaurs.. Journal of Vertebrate Paleontology.

[10] Wedel MJ (2003). The evolution of vertebral pneumaticity in sauropod dinosaurs.. Journal of Vertebrate Paleontology.

[11] Britt BB (1993). Pneumatic postcranial bones in dinosaurs and other archosaurs..

[12] Osborn HF, Mook CC (1921). *Camarasaurus*, *Amphicoelias*, and other sauropods of Cope.. Memoirs of the American Museum of Natural History (new series).

[13] Welles SP (1984). *Dilophosaurus wetherilli* (Dinosauria, Sauropoda) osteology and comparisons.. Palaeontographica.

[14] Lull RS, Wright NE (1942). Hadrosaurian dinosaurs of North America.. Geological Society of America Special Papers.

[15] Chatterjee S (1985). *Postosuchus*, a new thecodontian reptile from the Triassic of Texas and the origin of tyrannosaurs.. Philosophical Transactions of the Royal Society of London B.

[16] Camp CL (1930). A study of the phytosaurs with description of new material from western North America.. Memoirs of the University of California.

[17] Hatcher JB (1901). *Diplodocus* Marsh, its osteology, taxonomy, and probable habits, with a restoration of the skeleton.. Memoirs of the Carnegie Museum.

[18] Bonaparte JF (1999). Evolución de las vértebras presacras en Sauropodomorpha.. Ameghiniana.

[19] Harris JD (2006). The axial skeleton of the dinosaur *Suuwassea emilieae* (Sauropoda: Flagellicaudata) from the Upper Jurassic Morrison Formation of Montana, USA.. Palaeontology.

[20] Wilson JA, Upchurch P (2009). Redescription and reassessment of the phylogenetic affinities of *Euhelopus zdanskyi* (Dinosauria: Sauropoda) from the Early Cretaceous of China.. Journal of Systematic Palaeontology.

[21] González Riga BJ (2003). A new titanosaur (Dinosauria, Sauropoda) from the Upper Cretaceous of Mendoza Province, Argentina.. Ameghiniana.

[22] Curry Rogers K (2009). The postcranial osteology of *Rapetosaurus krausei* (Sauropoda: Titanosauria) from the Late Cretaceous of Madagascar.. Journal of Vertebrate Paleontology.

[23] Wedel MJ, Cifelli RL, Sanders RK (2000). Osteology, paleobiology, and relationships of the sauropod dinosaur *Sauroposeidon*.. Acta Palaentologica Polonica.

[24] Madsen JH, Welles SP (2000). *Ceratosaurus* (Dinosauria, Sauropoda) a revised osteology.. Utah Geological Survey Miscellaneous Publication.

[25] Madsen JH (1976). *Allosaurus fragilis*, a revised osteology.. Bulletin of the Utah Geological Survey.

[26] Whitlock JA, D'Emic MD, Wilson JA Cretaceous diplodocids in Asia? Re-evaluating the phylogenetic affinities of a fragmentary specimen.. Palaeontology. In press.

[27] Wilson JA (2006). Anatomical nomenclature of fossil vertebrates: standardized terms or lingua franca?. Journal of Vertebrate Paleontology.

[28] Salgado L, Powell JE (2010). Reassessment of the vertebral laminae in some South American titanosaurian sauropods.. Journal of Vertebrate Paleontology.

[29] Allain R, Aquesbi N (2008). Anatomy and phylogenetic relationships of *Tazoudasaurus naimi* (Dinosauria, Sauropoda) from the late Early Jurassic of Morocco.. GeoDiversitas.

[30] Allain R, Aquesbi N, Dejax J, Meyer C, Monbaron M (2004). A basal sauropod from the Early Jurassic of Morocco.. Comptes Rendus Palevol.

[31] Young CC (1954). On a new sauropod from Yiping, Szechuan, China.. Acta Scientia Sinica.

[32] Young CC, Chao S-C (1972). *Mamenchisaurus hochuanensis*, sp. nov.. Memoirs of the Institute of Vertebrate Palaeontology and Palaeoanthropology.

[33] Ouyang H, Ye Y (2001). The first mamenchisaurian skeleton with complete skull, *Mamenchisaurus youngi*..

[34] Wilson JA (2002). Sauropod dinosaur phylogeny: critique and cladistic analysis.. Zoological Journal of the Linnean Society.

[35] McIntosh JS, Miles CA, Cloward KC, Parker JR (1996). A new nearly complete skeleton of *Camarasaurus*.. Bulletin of Gunma Museum of Natural History.

[36] Ikejiri T, Tidwell V, Trexler DL, Tidwell V, Carpenter K (2005). New adult specimens of *Camarasaurus lentus* highlight variation ontogenetic variation within the species.. Thunder-lizards: the sauropodomorph dinosaurs.

[37] Cope ED (1877). On a gigantic saurian from the Dakota epoch of Colorado.. Paleontology Bulletin.

[38] Sereno PC, Wilson JA, Witmer LM, Whitlock JA, Maga A (2007). Structural extremes in a Cretaceous dinosaur.. PLoS ONE.

[39] O'Connor PM (2007). Postcranial axial skeleton of *Majungasaurus crenatissimus* (Theropoda: Abelisauridae) from the Late Cretaceous of Madagascar.. Society of Vertebrate Paleontology Memoir.

[40] Mcintosh JS, Weishampel DB, Dodson P, Osmólska H (1990). Sauropoda.. The Dinosauria.

[41] Paul GS (1988). The brachiosaur giants of the Morrison and Tendaguru with a description of a new subgenus, *Giraffatitan*, and a comparison of the world's largest dinosaurs.. Hunteria.

[42] Taylor MP (2009). A re-evaluation of *Brachiosaurus altithorax* Riggs 1903 (Dinosauria, Sauropoda) and its generic separation from *Giraffatitan brancai* (Janensch 1914).. Journal of Vertebrate Paleontology.

[43] Calvo JO, González Riga BJ (2003). *Rinconsaurus caudamirus* gen. et sp. nov., a new titanosaurid (Dinosauria, Sauropoda) from the Late Cretaceous of Patagonia, Argentina.. Revista Geologica de Chile.

[44] Suteethorn S, Le Loeuff J, Suteethorn V (2010). Description of topotypes of *Phuwiangosaurus sirindhornae*, a sauropod from the Sao Khua Formation (Early Cretaceous) of Thailand, and their phylogenetic implications.. Neues Jahrbuch für Geologie und Paläontologie Abhandlungen.

[45] Janensch W (1950). Die Wirbelsäule der von *Brachiosaurus brancai*.. Palaeontographica (Supplement 7).

[46] Curry Rogers K, Curry Rogers KA, Wilson JA (2005). Titanosauria.. The sauropods: evolution and paleobiology.

[47] Calvo JO, Porfiri J, González Riga BJ, Kellner AWA (2007). Anatomy of *Futalognkosaurus dukei* Calvo, Porfiri, González Riga & Kellner, 2007 (Dinosauria, Titanosauridae) from the Neuquén Group (Late Cretaceous), Patagonia, Argentina.. Arquivos do Museu Nacional, Rio de Janeiro.

[48] Gomani EM (2005). Sauropod dinosaurs from the Early Cretaceous of Malawi, Africa.. Palaeontologia Electronica.

[49] Curry Rogers K, Forster CA (2001). The last of the dinosaur titans: a new sauropod from Madagascar.. Nature.

[50] Campos DA, Kellner AWA, Bertini RJ, Santucci R (2005). On a titanosaurid (Dinosauria, Sauropoda) vertebral column from the Bauru Group, Late Cretaceous of Brazil.. Arquivos do Museu Nacional, Rio de Janeiro.

[51] Salgado L, García RA, Daza JD (2006). Consideraciones sobre las láminas neurales de los dinosaurios saurópodos y su significado morfofunctional.. Revista del Museo Argentino de Ciencias Naturales, nuevo series.

[52] Gilmore CW (1936). Osteology of *Apatosaurus* with special reference to specimens in the Carnegie Museum.. Memoirs of the Carnegie Museum.

[53] Upchurch P, Tomida Y, Barrett PM (2004). A new specimen of *Apatosaurus ajax* (Sauropoda: Diplodocidae) from the Morrison Formation (Upper Jurassic) of Wyoming USA.. National Science Museum Monographs.

[54] Sereno PC, Beck AL, Dutheil DB, Larsson HCE, Lyon GH (1999). Cretaceous sauropods from the Sahara and the uneven rate of skeletal evolution among dinosaurs.. Science.

[55] Whitlock JA A phylogenetic analysis of Diplodocoidea (Saurischia: Sauropoda).. Zoological Journal of the Linnean Society. In press.

[56] Coria RA, Salgado L (2000). A basal Abelisauria, Novas 1992 (Theropoda-Ceratosauria) from the Cretaceous of Patagonia, Argentina.. Gaia.

[57] Benson RBJ (2008). New information on *Stokesosaurus*, a tyrannosauroid (Dinosauria, Theropoda) from North America and the United Kingdom.. Journal of Vertebrate Paleontology.

[58] Farke AA (2010). Evolution and functional morphology of the frontal sinuses in Bovidae (Mammalia: Artiodactyla), and implications for the evolution of cranial pneumaticity.. Zoological Journal of the Linnean Society.

[59] Curry Rogers K, Forster CA (2004). The skull of *Rapetosaurus krausei* (Sauropoda, Titanosauria) from the Late Cretaceous of Madagascar.. Journal of Vertebrate Paleontology.

[60] Yates A (2007). The first complete skull of the Triassic dinosaur *Melanorosaurus haughton* (Sauropodomorpha: Anchisauria).. Special Papers in Paleontology.

[61] Holtz TR, Osmólska H, Weishampel DB, Dodson P, Osmólska H (2004). Saurischia.. The Dinosauria, 2nd Edition.

